# Substrate recognition by a bifunctional GH30‐7 xylanase B from *Talaromyces cellulolyticus*

**DOI:** 10.1002/2211-5463.12873

**Published:** 2020-05-22

**Authors:** Yusuke Nakamichi, Masahiro Watanabe, Akinori Matsushika, Hiroyuki Inoue

**Affiliations:** ^1^ Research Institute for Sustainable Chemistry National Institute of Advanced Industrial Science and Technology (AIST) Higashi‐Hiroshima Japan; ^2^ Graduate School of Integrated Sciences for Life Hiroshima University Higashi‐Hiroshima Japan

**Keywords:** crystal structure, enzyme–product complex, glucuronoxylanase, glycoside hydrolase family 30, *Talaromyces cellulolyticus*, xylobiohydrolase

## Abstract

Xylanase B, a member of subfamily 7 of the GH30 (glycoside hydrolase family 30) from *Talaromyces cellulolyticus* (*Tc*Xyn30B), is a bifunctional enzyme with glucuronoxylanase and xylobiohydrolase activities. In the present study, crystal structures of the native enzyme and the enzyme–product complex of *Tc*Xyn30B expressed in *Pichia pastoris* were determined at resolutions of 1.60 and 1.65 Å, respectively. The enzyme complexed with 2^2^‐(4‐*O*‐methyl‐α‐d‐glucuronyl)‐xylobiose (U^4m2^X) revealed that *Tc*Xyn30B strictly recognizes both the C‐6 carboxyl group and the 4‐*O*‐methyl group of the 4‐*O*‐methyl‐α‐d‐glucuronyl side chain by the conserved residues in GH30‐7 endoxylanases. The crystal structure and site‐directed mutagenesis indicated that Asn‐93 on the β2‐α2‐loop interacts with the non‐reducing end of the xylose residue at subsite‐2 and is likely to be involved in xylobiohydrolase activity. These findings provide structural insight into the mechanisms of substrate recognition of GH30‐7 glucuronoxylanase and xylobiohydrolase.

## Graphical abstract

*Tc*Xyn30B is a bifunctional xylanase with endo‐glucuronoxylanase and exo‐xylobiohydrolase activities. The present study determined the crystal structure of the *Tc*Xyn30B‐product complex. A 4‐*O*‐methyl‐α‐d‐glucuronyl moiety‐binding pocket required for glucuronoxylanase activity and Asn‐93 (which is likely involved in xylobiohydrolase activity) was structurally characterized. The present study provides insight into the mechanisms of substrate recognition of a bifunctional xylanase.

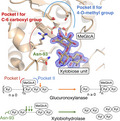

**Abbreviations**
GH30‐7: subfamily 7 of the glycoside hydrolase family 30
GH30‐8: subfamily 8 of the glycoside hydrolase family 30
MeGlcA: α‐1,2‐linked‐4‐*O*‐methyl‐d‐glucuronic acid
U^4m2^X: 2^2^‐(4‐*O*‐methyl‐α‐d‐glucuronyl)‐xylobiose
X_3_: xylotriose
X_n_U^4m2^X: 2^2^‐MeGlcA‐xylooligosaccarides
XU^4m2^X: 2^2^‐MeGlcA‐xylotriose

Xylan is the major component of hemicellulose in plants. Xylan is composed of a linear backbone of β‐d‐xylopyranosyl residues linked by β‐1,4‐glycosidic bonds, which are further decorated with side‐chain residues, such as α‐1,2‐ and/or α‐1,3‐linked‐l‐arabinofuranose, and α‐1,2‐linked‐4‐*O*‐methyl‐d‐glucuronic acid (MeGlcA). Glucuronoxylanase (https://www.qmul.ac.uk/sbcs/iubmb/) is an appendage‐dependent endoxylanase that must recognize an α‐1,2‐linked MeGlcA common to glucuronoxylans for hydrolysis. Glucuronoxylanase cleaves the glucuronoxylan main chain at the second glycosidic linkage from the MeGlcA substituent toward the reducing end to produce 2^2^‐MeGlcA‐xylooligosaccarides (X_n_U^4m2^X, *n* ≥ 0). The enzyme is classified into glycoside hydrolase family (GH) 30 subfamilies 7 and 8 (GH30‐7 and 30‐8) in the CAZy database (http://www.cazy.org) [1].

Typically, subfamily 8 of the glycoside hydrolase family 30 (GH30‐8) glucuronoxylanases primarily occur in bacteria [2, 3, 4, 5]. Crystal structures of GH30‐8, such as *Ec*XynA from *Dickeya chrysanthemi* (formerly *Erwinia chrysanthemi*) and *Bs*XynC from *Bacillus subtilis*, have revealed that the enzymes consist of a (β/α)_8_‐barrel with an obligatory side‐associated, nine‐stranded, aligned β‐sandwich [1, 6]. This side β‐sandwich structure is tightly associated with the (β/α)_8_‐barrel catalytic core domain. Studies of ligand‐bound GH30‐8 xylanase structures have identified the role of the β7–α7 and β8–α8 loop regions in the specific coordination of the MeGlcA substituent through a salt bridge established between the C‐6 carboxylate of the MeGlcA and an arginine (Arg‐293 of *Ec*XynA and Arg‐272 of *Bs*XynC) that extends from the β8–α8 loop (Fig. 1) [7, 8, 9].

**Fig. 1:**
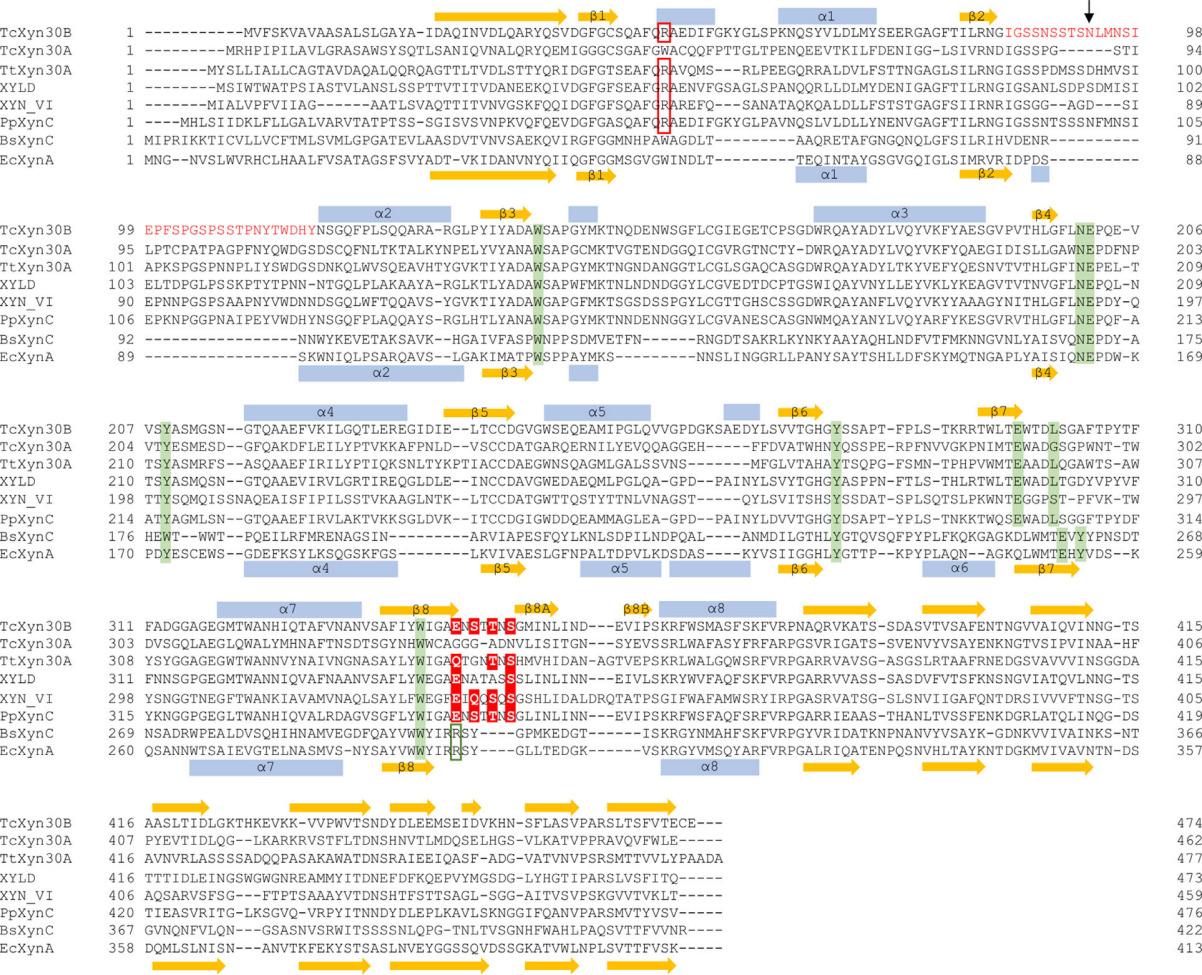
Multiple sequence alignment of GH30‐7 and GH30‐8 xylanases. Primary structures of *Tc*Xyn30B (NCBI accession ID, GAM36763), *Tc*Xyn30A from *T. cellulolyticus* (GAM43270), *Tt*Xyn30A from *T. thermophila* (XP_003660270), XYLD from *Bispora* sp. MEY‐1 (ADG62369), XYN VI from *T. reesei* (EGR45006), *Pp*XynC from *P. purpurogenum* (AKH40280), *Bs*XynC from *B. subtilis* (CAA97612) and *Ec*XynA from *D. chrysanthemi* (formerly *E. chrysanthemi*) (AAB53151) were used for sequence alignment. The features shown are: α‐helices of *Tc*Xyn30B (upper) and *Ec*XynA (lower) (blue boxes); β‐strands of *Tc*Xyn30B (upper) and *Ec*XynA (lower) (yellow arrows); Arg residue conserved in GH30‐7 glucuronoxylanases and endoxylanases (boxed by red lines); the β2‐α2 loop of *Tc*Xyn30B (red letters); the position of Asn‐93 of *Tc*Xyn30B (a black arrow); residues composed of subsites ‐1 (highlighted in green); Arg residue conserved in GH30‐8 glucuronoxylanases (boxed by a green line); and the conserved residues composing the recognition pocket of 4‐*O*‐methyl‐group (highlighted in red).

GH30‐7 glucuronoxylanases have been found in fungi, such as XYN VI from *Trichoderma reesei*, Xyn30B from *Talaromyces cellulolyticus* (*Tc*Xyn30B) and Xyn30A from *Thermothelomyces thermophila* (*Tt*Xyn30A) [10, 11, 12]. Unlike the GH30‐8 enzyme, these enzymes act on unsubstituted xylan and xylooligosaccharides. Especially, *Tc*Xyn30B and *Tt*Xyn30A have been reported as bifunctional xylanases possessing both glucuronoxylanase and xylobiohydrolase activities, which release xylobiose from non‐reducing ends of X_n_U^4m2^X (*n* ≥ 0) produced by glucuronoxylanase activity [11, 12].

We recently determined the 3D‐structure of *Tc*Xyn30B as the first structure of a GH30‐7 xylanase [11]. The overall structure of *Tc*Xyn30B is basically similar to GH30‐8 enzymes. In addition, *Tc*Xyn30B has unique structural features, which are probably conserved in other GH30‐7 enzymes. They include a Cys‐pair (*cis*‐Cys‐241 and Cys‐242); a β8‐sheet consisting of strands β8, β8A and β8B; and no α6 helix (Fig. 1) [11]. X‐ray crystallography and mutational analysis of *Tc*Xyn30B without any ligands have suggested that Arg‐46 from the β1‐α1 region conserved in GH30‐7 endoxylanases plays a critical role in recognizing MeGlcA for glucuronoxylanase activity [11]. We also predict that Asn‐93 in the β2–α2 loop may contribute to xylobiohydrolase activity, using the *Tc*Xyn30B structure that was superimposed on the GH30‐8 *Ec*XynA model complexed with 2^2^‐MeGlcA‐xylotriose (XU^4m2^X) [8]. However, structural factors for substrate recognition cannot be fully explained because the amino acid sequence identity between *Tc*Xyn30B and *Ec*XynA is low (24%). Especially, residues involved in the recognition of MeGlcA of GH30‐8 enzymes are not conserved in GH30‐7 enzymes including *Tc*Xyn30B. It is also unclear how Asn‐93 in the loop actually interacts with the substrate. In the present study, the crystal structure of *Tc*Xyn30B complexed with 2^2^‐MeGlcA‐xylobiose (U^4m2^X) is determined. U^4m2^X is a minimum product obtained by glucuronoxylanase activity and an appropriate ligand for understanding the recognition mechanism for MeGlcA and xylobiose. Structural analysis of *Tc*Xyn30B‐U^4m2^X provides valuable insights into the catalytic properties of GH30‐7 bifunctional glucuronoxylanase and xylobiohydrolase.

## Materials and methods

### Expression of recombinant *Tc*Xyn30B

Recombinant *Tc*Xyn30B was expressed in *Pichia pastoris* using the *Pichia* Expression Kit (Thermo Fisher Scientific, Waltham, MA, USA). The pPIC9K plasmid (Thermo Fisher Scientific) was used to construct an expression plasmid to produce *Tc*Xyn30B. *Escherichia coli* DH5α (TaKaRa Bio, Kyoto, Japan) was used for the DNA procedures. The *Tc*Xyn30B gene excluding signal sequence (residues 1–22) was synthesized. The *xyn30B* gene coding residues 23–474 was amplified using the forward primer, 5'‐GAATTCCAGATTAATGTGGATCTGCAAGCTCGC‐3', with the *Eco*RI site (underlined) and the reverse primer, 5'‐GCGGCCGCTCATTCGCATTCGGTCACAAAGCTGG‐3', with the *Not*I site (underlined). The expression plasmid, pPIC9K‐*Tc*Xyn30B, was constructed by ligating the *xyn30B* fragment that had been digested with *Eco*RI/*Not*I into the corresponding site of pPIC9K. The presence of the ligated gene fragment and its location were confirmed by DNA sequencing.

Recombinant *Tc*Xyn30B with eight His‐tag at the C‐terminal (*Tc*Xyn30B‐His) and its mutant, *Tc*Xyn30B‐His N93A, were expressed using the same procedure as described above. The expression plasmid, pPIC9K‐*Tc*Xyn30B‐His, was constructed by site‐directed mutagenesis of pPIC9K‐*Tc*Xyn30B using the KOD ‐plus‐ Mutagenesis kit (Toyobo, Osaka, Japan). The forward primer 5'‐CATCATCACCATCACCACCATCACTGAGCGGCCGCGAATTAATTCGC‐3' (insertion region underlined) and the reverse primer, 5'‐TTCGCATTCGGTCACAAAGCTGGTCA‐3', were used for PCR. The expression plasmid, pPIC9K‐*Tc*Xyn30B‐His N93A, was constructed by site‐directed mutagenesis of pPIC9K‐*Tc*Xyn30B‐His. The forward primer 5'‐GCTTTAATGAACAGCATTGAGCCGTTTAGC‐3' (mutation site underlined) and the reverse primer, 5'‐GCTGGTGCTGCTATTGCTGCTGCCGATGCC‐3', were used for PCR. The presence of all ligated gene fragments and their locations were confirmed by DNA sequencing.

The pPIC9K‐*Tc*Xyn30B, pPIC9K‐*Tc*Xyn30B‐His and pPIC9K‐*Tc*Xyn30B‐His N93A were linearized by *Sac*I and transformed into *P. pastoris* GS115 (Thermo Fisher Scientific) by electroporation. The strains producing *Tc*Xyn30B, *Tc*Xyn30B‐His, and *Tc*Xyn30B‐His N93A were selected based on the amount of recombinant protein in culture supernatant as visualized by SDS/PAGE using NuPage 4–12% Bis‐Tris gels (Invitrogen, Carlsbad, CA, USA). To produce recombinant proteins, the selected strains were cultured in a BMMY medium (1% yeast extract, 2% peptone, 100 mm potassium phosphate, pH 6.0, 1.34% yeast nitrogen base, 4 × 10^−5^% biotin and 0.5% methanol) as described in the manufacturer’s instructions for the *Pichia* Expression Kit (Thermo Fisher Scientific).

### Purification of *Tc*Xyn30B, *Tc*Xyn30B‐His, and *Tc*Xyn30B‐His N93A

Purification of *Tc*Xyn30B, *Tc*Xyn30B‐His and *Tc*Xyn30B‐His N93A was performed using an ÄKTA purifier chromatography system (GE Healthcare, Little Chalfont, UK) at room temperature. A culture supernatant including *Tc*Xyn30B was filtered through a 0.22‐μm polyethersulfone membrane and the filtrate protein was concentrated and changed to 20 mm 2‐(*N*‐morpholino) ethanesulfonic acid (pH 6.0) using a Vivaspin 20‐10K centrifugal concentrator (Sartorius, Göttingen, Germany). The sample was applied to a HitrapQ anion‐exchange column (5 mL; GE Healthcare) that had been equilibrated with the same buffer, and protein peaks were eluted with a linear gradient of 0–0.5 m NaCl (20 column volumes) at a flow rate of 2 mL·min^–1^. Fractions containing the target proteins were confirmed by SDS/PAGE and pooled. (NH_4_)_2_SO_4_ was added to a final concentration of 2.0 m and then the samples were subjected to ResourceISO (6 mL; GE Healthcare) hydrophobic interaction chromatography using a 2.0–0 m (NH_4_)_2_SO_4_ gradient (20 column volumes) in 20 mm sodium acetate buffer (pH 4.0) at a flow rate of 1 mL·min^−1^. The fractions containing target protein were pooled and concentrated by ultrafiltration using a Vivaspin 20‐5K centrifugal concentrator. The sample was applied to a Superdex 200 Increase 10/300 GL size exclusion chromatography column (GE Healthcare) that had been equilibrated with 0.15 m NaCl in 20 mm sodium acetate buffer (pH 4.0).

Culture supernatants including *Tc*Xyn30B‐His and *Tc*Xyn30B‐His N93A were mixed with Tris‐HCl (pH 8.0) at a final concentration of 50 mm and then filtered through a 0.22‐μm polyethersulfone membrane. The samples were applied to a HisTrap FF Ni‐affinity column (10 mL; GE Healthcare) that had been equilibrated with 20 mm imidazole in 20 mm Tris‐HCl (pH 7.5) and the column was washed using 40 mm imidazole. Protein peaks were eluted with a linear gradient of 40–300 mm imidazole (20 column volumes) at a flow rate of 4 mL·min^−1^. Fractions containing the target proteins were confirmed by SDS/PAGE and pooled. (NH_4_)_2_SO_4_ was added to final concentration of 2.0 m and the samples were then subjected to HiTrap Butyl HP (5 mL; GE Healthcare) hydrophobic interaction chromatography using a 2.0–0 m (NH_4_)_2_SO_4_ gradient (20 column volumes) in 20 mm sodium acetate buffer (pH 4.0) at a flow rate of 4 mL·min^−1^.

All purified enzymes were preserved in a 20 mm sodium acetate buffer (pH 4.0) at 4 °C. Protein concentration was determined by monitoring *A*_280_.

### Mass spectrometry

The molecular weight of the purified *Tc*Xyn30B was evaluated by MALDI time‐of‐flight MS with a Spiral TOF JMS‐S3000 (JEOL, Tokyo, Japan) as described previously [11]. The purified sample was applied to the MALDI target plate after dilution into a mixture containing 0.5% (w/v) sinapinic acid, 0.1% trifluoroacetic acid and 25% acetonitrile.

### X‐ray crystallography

Purified *Tc*Xyn30B was concentrated to 10 mg·mL^−1^ for crystallization by ultrafiltration using a Vivaspin 20‐5K centrifugal concentrator. Crystals were obtained with the hanging‐drop vapor diffusion method at 20 °C for 1 week. The drop was comprised 1.0 µL of protein solution mixed with 1.0 µL of reservoir solution containing 25% poly(ethylene glycol) 3350, 0.1 m Hepes‐sodium hydroxide (pH 7.5) and 200 mm magnesium chloride and was equilibrated against 500 µL of reservoir solution. In the case of the co‐crystallization with a ligand, the 2.0‐µL drops were prepared by mixing the protein, ligand and precipitant solutions at a volume ratio of 0.9 : 0.1 : 1. A mixture of aldouronic acids (Megazyme, Wicklow, Ireland) containing a mixture of U^4m2^X, 2^3^‐MeGlcA‐xylotriose (U^4m2^XX) and 2^4^‐MeGlcA‐xylotetraose (U^4m2^XXX) at a ratio of 2 : 2 : 1 was used as the ligand solution. The abbreviations used to describe the xylooligosaccharides have been reported previously [13]. The structures of ligands are shown in Fig. S1. A mixture containing 25% poly(ethylene glycol) 3350, 0.1 m Hepes‐sodium hydroxide (pH 7.3) and 200 mm magnesium chloride was used as a precipitant solution for co‐crystallization.

The crystals of *Tc*Xyn30B and the enzyme complexed with the mixture of aldouronic acids were soaked with the reservoir solution supplemented with 25% (v/v) glycerol and 10% (w/v) poly(ethylene glycol) 3350 as cryo‐protectants, respectively, and then flash cooled in liquid nitrogen. X‐ray diffraction data of crystals of *Tc*Xyn30B and *Tc*Xyn30B complexed with U^4m2^X were collected to resolutions of 1.60 and 1.65 Å at 100 K at the SPring‐8 beamline BL44XU (Hyogo, Japan). Diffraction images were checked with adxv (http://www.scripps.edu/tainer/arvai/adxv.html) and integrated and scaled with xds (version: 15 March 2019) [14]. Phasing was performed using molrep, version 11.6, in ccp4, version 7.0, with *Tc*Xyn30B coordinates (PDB ID: http://6IUJ) as the model [15, 16]. The model was manually completed using coot, version 0.8.9 [17], and refined using Phenix.refine [18] in phenix, version 1.12 [19], and refmac, version 5.8 [20]. Model quality was verified using molprobity, version 4.4 [21]. Superpositioning of protein models and calculation of their rmsd were conducted using LSQKAB program in ccp4 program package [22]. Molecular figures were generated with pymol, version 1.8 (Schrödinger, LLC, New York, NY, USA).

### Enzyme assays

All assays were performed in triplicate. Glucuronoxylanase activity was measured by assaying the reducing sugars released after the enzyme reaction with 10 mg·mL^–1^ beechwood glucuronoxylan (Megazyme) using 3,5‐dinitrosalicylic acid. The enzyme reaction was performed under conditions of 50 mm sodium acetate buffer (pH 4.0) at 40 °C for 15 min. One unit of glucuronoxylanase activity was defined as the amount of protein that could yield 1 μmol of reducing sugar per minute from the hydrolysis of beechwood glucuronoxylan.

Xylobiohydrolase activity was measured in a reaction mixture containing 2 mm xylotriose (X_3_; Megazyme) in 50 mm sodium acetate (pH 4.0). The reaction was carried out at 40 °C for 15 min. The released xylose was analyzed by high‐performance anion‐exchange chromatography with pulsed amperometric detection using a Dionex ICS‐3000 ion chromatography system (Dionex, Sunnyvale, CA, USA) [23]. One unit of xylobiohydrolase activity for X_3_ was defined as the amount of protein that could release 1 μmol xylose·min^–1^.

Determination of the kinetic parameters of *Tc*Xyn30B‐His and *Tc*Xyn30B‐His N93A was performed using 3.6–48 mg·ml^−1^ beechwood glucuronoxylan and 1–16 mm X_3_. The reaction was performed at 40 °C in 50 mm sodium acetate buffer (pH 4.0). Kinetic constants for beechwood glucuronoxylan were determined using the nonlinear least‐squares data fitting method in excel, version 2016 (Microsoft Corp., Redmond, WA, USA) [24]. The initial slopes of the progress curves were used to determine the catalytic efficiency (*k*_cat_/*K*_m_) of X_3_. All assays were carried conducted in triplicate.

## Results and Discussion

### Expression, purification, and crystallization

The *Tc*Xyn30B protein was overexpressed and secreted extracellularly by *P. pastoris* expression system. *Tc*Xyn30B was purified to homogeneity (Fig. S2). The average molecular mass of *Tc*Xyn30B from *P. pastoris* was determined as 62 182 Da by time‐of‐flight MS. This value was significantly higher than that of *Tc*Xyn30B (56 354 Da) produced using the *T. cellulolyticus* homologous expression system [11], meaning that glycosylation patterns between two proteins are different. The glycosylation patterns of *Tc*Xyn30B from *P. pastoris* used in the present study were assigned by X‐ray crystallography, as described below. Crystals of ligand‐free *Tc*Xyn30B were obtained by hanging‐drop vapor diffusion. Crystals of the *Tc*Xyn30B–U^4m2^X complex were obtained by a co‐crystallization method under almost the same conditions as those used for the ligand‐free crystals (Fig. S3).

### Structure determination

**Fig. 2:**
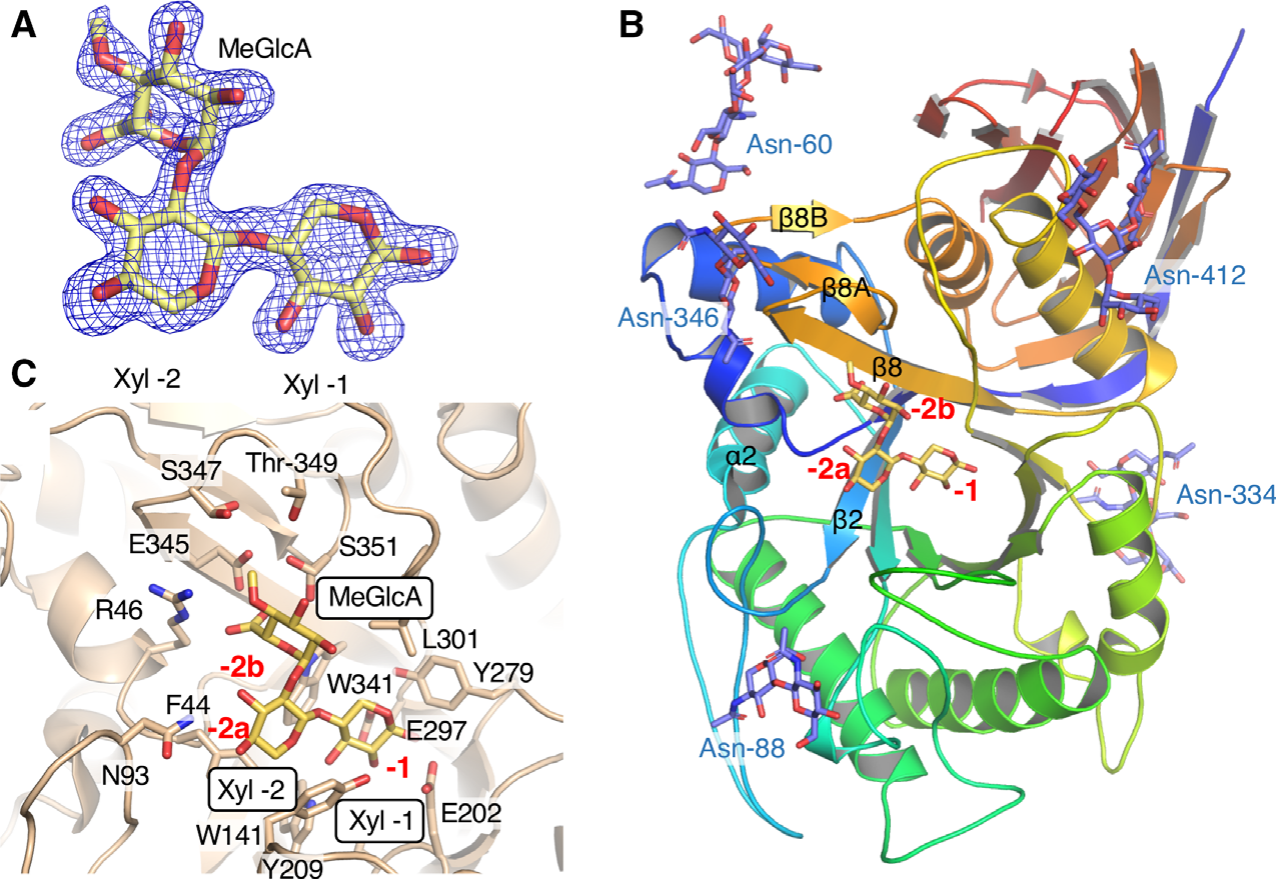
The structure of *Tc*Xyn30B complexed with a ligand. (A) *F*_o_‐*F*_c_ omit maps (blue) contoured at 3.0 σ for U^4m2^X in the crystals of *Tc*Xyn30B with a ligand. Two Xyl residues with the MeGlcA moiety are bound in subsites ‐1, and ‐2. (B) Overall structure of *Tc*Xyn30B (ribbon model) with U^4m2^X (yellow stick model) and *N*‐linked sugar chains (purple stick model). The ribbon is coloured from the N terminus to the C terminus in a progression from blue to red. Black lettering indicates the positions of β2‐strand, α2‐helix and β8‐sheet (composed of β8, β8A, and β8B). Red numbers show subsites. Positions of glycosylated‐Asn residues and *N*‐linked sugar chains are shown in blue. (C) The active site structure of *Tc*Xyn30B complexed with U^4m2^X.

Both ligand‐free and ligand‐complexed *Tc*Xyn30B crystals belonged to the *P*2_1_2_1_2_1_ space group. Diffraction data statistics are shown in Table 1. The crystal structures of ligand‐free and ligand‐complexed enzymes were determined at resolutions of 1.60 and 1.65 Å, respectively, by molecular replacement, using *Tc*Xyn30B from *T. cellulolyticus* as the search model (PDB ID: http://6IUJ). One protein molecule was contained in an asymmetric unit. Amino acid residues numbered 20–473 and 18–473 for *Tc*Xyn30B without and with ligand were assigned with the electron density map, respectively. Amino acid residues 18‐22 (AYVEF) are from DNA sequence included in pPIC9K vector, whereas residues 23 or later are numbered as with native protein. U^4m2^X was modeled at later stages of refinement, when the electron density was unambiguous (Fig. 2A). The overall structure of ligand‐free *Tc*Xyn30B from *P. pastoris* was almost the same as that of the *Tc*Xyn30B‐U^4m2^X complex (0.131 Å rmsd over 447 Cα atoms) by a least‐squares superposition method [22]. Similarly, there was no difference between the ligand‐free 3D‐structures of *Tc*Xyn30B from *P. pastoris* and *T. cellulolyticus* (0.550 Å rmsd over 447 Cα atoms).

**Table 1:** Statistics for X‐ray crystallography.

	*Tc*Xyn30B	*Tc*Xyn30B with U^4m2^X
Data collection		
Wavelength (Å)	0.9	0.9
Resolution range (Å)	43.14–1.60 (1.66–1.60)^a^	33.41–1.65 (1.71–1.65)
Space group	*P*2_1_2_1_2_1_	*P*2_1_2_1_2_1_
Unit cell		
*a*, *b*, *c* (Å)	63.34, 78.77, 117.84	63.25, 78.70, 118.42
Total reflections	526 004 (50 937)	317 528 (31 543)
Unique reflections	78 001 (7458)	71 087 (7027)
Multiplicity	6.7 (6.8)	4.5 (4.5)
Completeness (%)	99.6 (96.6)	99.5 (99.0)
Mean *I*/σ(*I*)	11.78 (2.00)	17.18 (2.55)
Wilson *B*‐factor	21.1	22.3
*R*‐merge	0.093 (0.801)	0.046 (0.458)
*R*‐pim	0.039 (0.332)	0.024 (0.239)
CC_1/2_	0.996 (0.660)	0.999 (0.847)
Refinement		
Reflections used in refinement	77 992 (7458)	71 083 (7027)
Reflections used for *R*‐free	3900 (373)	3554 (351)
*R*‐work	0.180 (0.334)	0.170 (0.242)
*R*‐free	0.202 (0.320)	0.193 (0.267)
CC (work)	0.957 (0.743)	0.957 (0.827)
CC (free)	0.950 (0.743)	0.961 (0.853)
Number of non‐hydrogen atoms	4175	4306
Macromolecules	3578	3559
Sugar chains and ligands	187	249
Solvent	410	498
Protein residues	454	456
rms (bonds)	0.014	0.007
rms (angles)	1.84	1.20
Ramachandran plot		
Favoured (%)	95.58	95.81
Allowed (%)	3.98	3.74
Outliers (%)	0.44	0.44
Average *B*‐factor	24.5	25.9
Macromolecules	23.1	24.4
Sugar chains and ligands	31.7	34.1
Solvent	33.4	33.0
PDB ID	http://6KRL	http://6KRN

^a^ Values in parentheses are for the highest resolution shell.

In the electron density maps, *N*‐glycosylation of *Tc*Xyn30B from *P. pastoris* is observed at Asn‐60, Asn‐88, Asn‐334, Asn‐346 and Asn‐412 (Figs 2B and S4), whereas *Tc*Xyn30B from *T. cellulolyticus* is glycosylated at Asn‐60, Asn‐88, Asn‐215, Asn‐334, Asn‐346 and Asn‐412 [11]. This is probably a result of differences in glycosylation mechanisms in the expression hosts. Comparison of the length of the sugar chain suggests that protein expressed by *P. pastoris* tends to possess a larger degree of polymerization than that expressed by *T. cellulolyticus*, although all sugar chains could not be assigned by electron density maps.

### Substrate recognition at subsite ‐2b

**Fig. 3:**
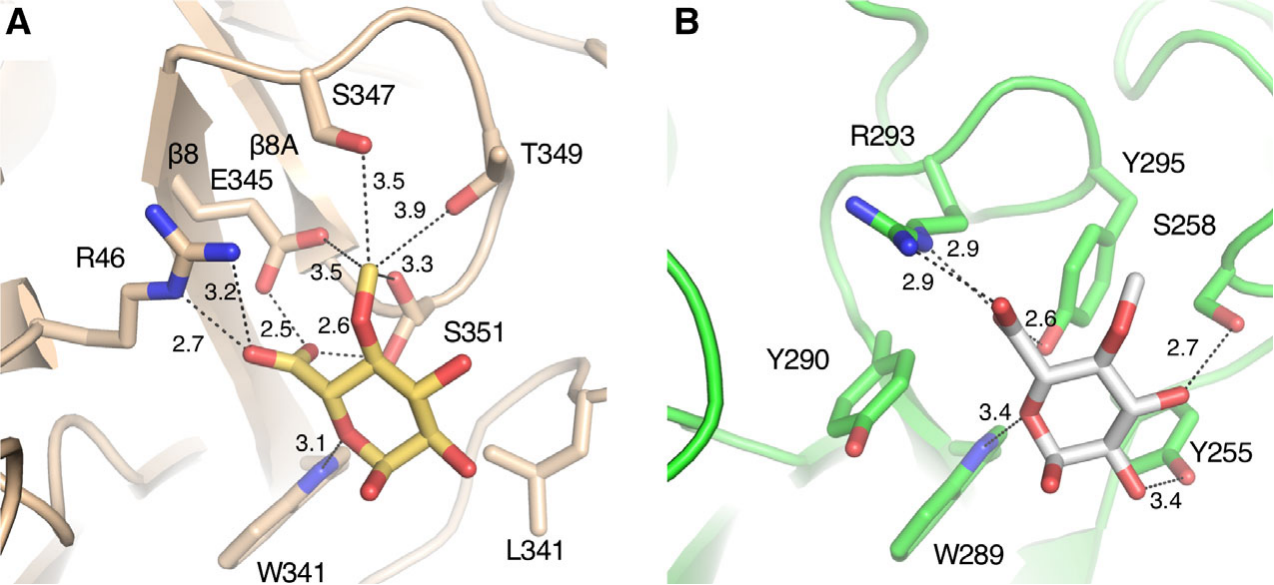
Detailed view of the interaction of the enzymes with MeGlcA derived from the *Tc*Xyn30B with U^4m2^X (A) and *Ec*XynA with XU^4m2^X (PDB ID: http://2Y24) (B). Amino acids and ligands are stick representations. Atoms are coloured as: C of *Tc*Xyn30B, brown; C of U^4m2^X, yellow; C of *Ec*XynA, green; C of XU^4m2^X bound to *Ec*XynA, white; O, red; N, blue. Relevant interatomic distances (Å) are indicated by dashed lines.

A clear density map for U^4m2^X is observed in the active cleft (Fig. 2A). Two xylose units modeled in subsites ‐1 and ‐2a are named Xyl ‐1 and Xyl ‐2, respectively. MeGlcA is bound in subsite ‐2b (Fig. 2C). The subsite ‐2b is composed of seven amino acid residues (Fig. 3A). The side chains of five amino acid residues are concentrated near the C‐6 carboxyl group and the 4‐*O*‐methyl group of the MeGlcA substituent. The C‐6 carboxyl group of MeGlcA is suggested to form hydrogen bonds with Glu‐345 and Ser‐351. Arg‐46 appears to form salt bridge with the C‐6 carboxyl group, similar to an Arg residue conserved in GH30‐8 glucuronoxylanase (Arg‐293 of *Ec*XynA) (Fig. 3A,B) [7, 8], in agreement with our previous prediction using a superimposed model structures of Xyn30B based on the *Ec*XynA model complexed with XU^4m2^X [11].

The side chains of Glu‐345, Ser‐347, Thr‐349 and Ser‐351 from β8 and a β8‐β8A loop are located near the 4‐*O*‐methyl group of MeGlcA (Figs 2C and 3A). The distances between the C‐atom of the 4‐*O*‐methyl group and the O‐atoms of Glu‐345, Ser‐347, Thr‐349 and Ser‐351 are 3.5, 3.5, 3.9 and 3.3 Å, respectively, suggesting that a part of these residues and the methyl group may form C‐H…O type of hydrogen bonds, which is a common but underappreciated interaction in biomolecules and molecular recognition (Fig. 3A) [25]. Glu‐345, which corresponded to Arg‐293 of *Ec*XynA, and Ser‐351 are highly conserved in other GH30‐7 endoxylanases (Fig. 1, highlighted in red) and are considered to play an important role in the recognition of both the C‐6 carboxyl and 4‐*O*‐methyl groups of MeGlcA. Moreover, Ser‐347 and Thr‐349 of *Tc*Xyn30B are partially conserved with polar residues in *Tt*Xyn30A, XYN VI and *Penicillium purpurogenum* XynC endoxylanase (*Pp*XynC) (Fig. 1, highlighted in red). By contrast, *Ec*XynA and *Bs*XynC, which lack a β8‐sheet structure composed of β8, β8A and β8B, have no structure involved in the recognition of a 4‐*O*‐methyl‐group [7, 8]. *Ec*XynA displays almost equivalent activity towards beechwood xylan and 4‐deoxy‐hexenuronosyl beechwood xylan, in which the methyl esters on the 4‐*O*‐methyl glucuronic acid substituents are removed [26]. These observations suggest that a methyl‐group recognition pocket is a unique feature of GH30‐7 endoxylanases with the β8‐sheet structure.

The orientation of the MeGlcA moiety bound to *Tc*Xyn30B is different from that of the moiety bound to *Ec*XynA (Fig. S5). The shifts of the C‐6 carboxyl groups and the methyl‐groups between MeGlcA moieties in two enzymes are 2.6 and 2.0 Å, respectively (Fig. S5), indicating that interactions of *Tc*Xyn30B with two functional groups significantly influence the substrate position.

### Substrate recognition at subsite ‐1 and ‐2a

**Fig. 4:**
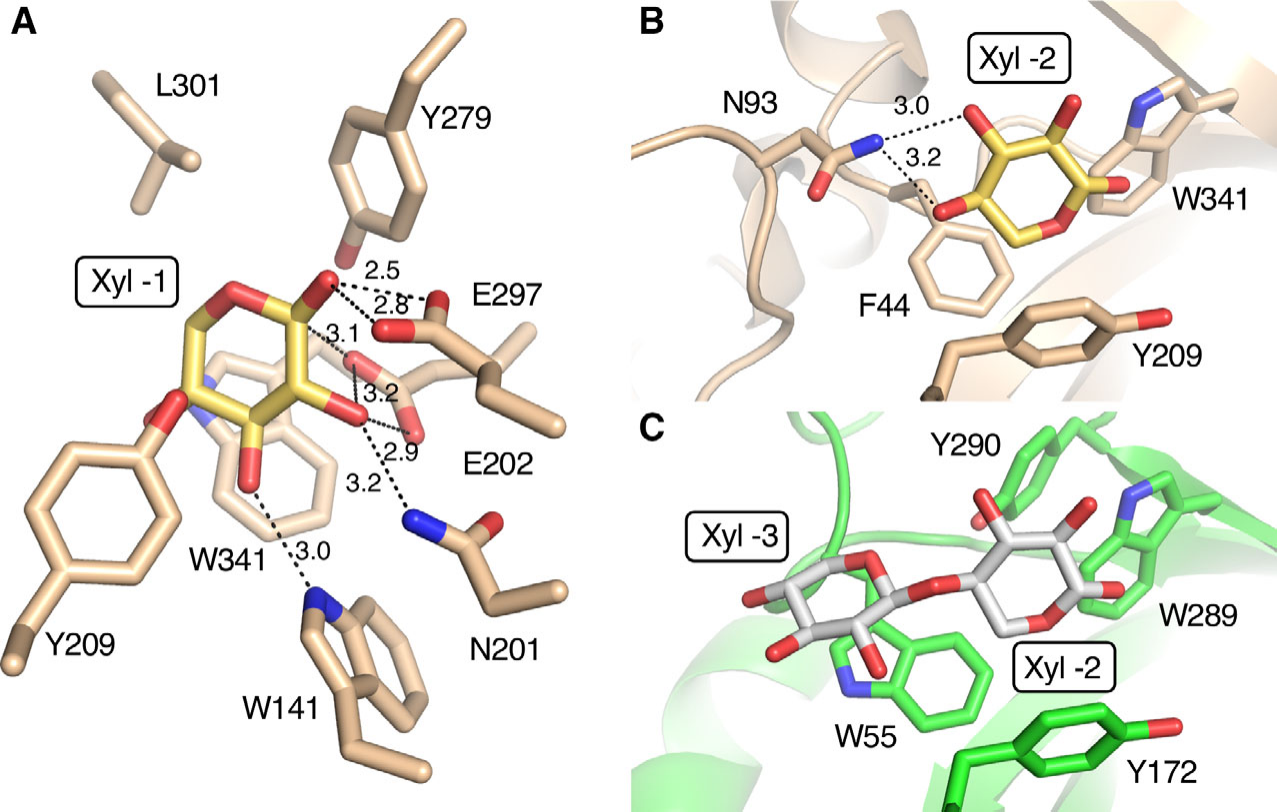
Detailed view of the interaction of the enzymes with xylose residues at negative subsites derived from the *Tc*Xyn30B with U^4m2^X (A, B) and *Ec*XynA with XU^4m2^X (PDB ID: http://2Y24) (C). (A) Subsite ‐1 of *Tc*Xyn30B. (B) Subsite ‐2 of *Tc*Xyn30B. (C) Subsite ‐2 and ‐3 of *Ec*XynA. Models are illustrated as in Fig. 3.

The subsite ‐1 of *Tc*Xyn30B is composed of Trp‐141, Asn‐201, Glu‐202, Tyr‐209, Tyr‐279, Glu‐297, Leu‐301 and Trp‐341 (Fig. 4A). All of these residues except Leu‐301 are conserved in both GH30‐7 and GH30‐8 (Fig. 1, highlighted in green).

At subsite ‐2a of *Tc*Xyn30B, the Xyl ‐2 residue takes part in the stacking interaction with the aromatic ring of Tyr‐209 and hydrophobically interacts with Phe‐44 and Trp‐341, similarly to the Xyl ‐2 residue in the *Ec*XynA that takes part in the interaction with Tyr‐172, Trp‐55 and Trp‐289 (Fig. 4B,C). Asn‐93 in the β2‐α2 loop is a notable residue that is not observed in GH30‐8 xylanases (Figs 2C and 4B,C). The distances between the O3 and O4 atoms of Xyl ‐2 and the Nδ atom of Asn‐93 in *Tc*Xyn30B are 3.0 and 3.2 Å, respectively (Fig. 4B). This suggests that the xylobiohydrolase activity found in *Tc*Xyn30B can be attributed to the interaction between Xyl ‐2 at the non‐reducing end and Asn‐93. Xylobiohydrolase activity has also been reported in *Tt*Xyn30A [12]. *Pp*XynC endoxylanase releases xylobiose from linear xylooligosaccharides [27]. These two enzymes have Asp and Asn residues, respectively, corresponding to Asn‐93 of *Tc*Xyn30B (Fig. 1) and these residues may play a similar role to Asn‐93 of *Tc*Xyn30B with respect to the release of xylobiose. On the other hand, Asn‐93 does not appear to be conserved in *Bispora* sp. MEY‐1 XYLD endoxylanase, *T. cellulolyticus* Xyn30A exoxylanase (*Tc*Xyn30A) and XYN VI glucuronoxylanase [10, 23, 28]. Xyn30A and XYN VI possess shorter β2‐α2 loops than that of *Tc*Xyn30B (Fig. 1). The β2‐α2 loop of *Tc*Xyn30A was predicted to not protrude into the active site by homology modeling [23].

To evaluate the role of Asn‐93 in *Tc*Xyn30B, His‐tagged *Tc*Xyn30B (*Tc*Xyn30B‐His) and its mutant enzyme whose Asn‐93 was replaced by Ala (*Tc*Xyn30B‐His N93A) were prepared. The glucuronoxylanase activities of *Tc*Xyn30B‐His and *Tc*Xyn30B‐His N93A for beechwood xylan were 10.9 ± 0.5 and 12.0 ± 0.2 U·mg^−1^, respectively. By contrast, the xylobiohydrolase activities of *Tc*Xyn30B‐His and *Tc*Xyn30B‐His N93A for X_3_ were 0.290 ± 0.006 and 0.0987 ± 0.004 U·mg^−1^, respectively. The kinetic parameters for each enzyme activity of *Tc*Xyn30B‐His and *Tc*Xyn30B‐His N93A are shown in Table 2. The *K*_m_ and *k*_cat_ values for the xylobiohydrolase activity could not be determined because the initial rate of xylose production from X_3_ was not saturated even at a substrate concentration of 16 mm. However, the three‐fold reduction of the *k*_cat_/*K*_m_ value in the N93A mutant suggests that Asn‐93 could contribute to increased catalytic efficiency for xylobiohydrolase activity but is not essential. These results also support the hypothesis that Asn‐93 interacts with the non‐reducing end of xylose residue at the subsite ‐2.

**Table 2:** Kinetic parameters of *Tc*Xyn30B‐His and *Tc*Xyn30B‐His N93A. ND, not determined because *K*_m _>> [S].

Enzymes	Beechwood glucuronoxylan			X_3_		
	*K*_m_ (mg·mL^−1^)	*k*_cat_ (s^−1^)	*k*_cat_/*K*_m_ (s^−1^·mg^−1^ mL)	*K*_m_ (mg)	*k*_cat_ (s^−1^)	*k*_cat_/*K*_m_ (s^−1^·mm ^ −1^)
*Tc*Xyn30B‐His	22.8 ± 0.6	24.4 ± 0.9	1.07 ± 0.05	ND	ND	0.144 ± 0.010
*Tc*Xyn30B‐His N93A	20.0 ± 1.8	23.2 ± 0.8	1.16 ± 0.10	ND	ND	0.0517 ± 0.0017

**Fig. 5:**
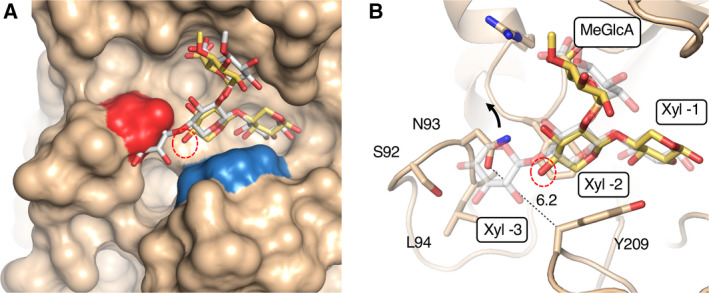
The *Tc*Xyn30B model complexed with U^4m2^X is superimposed on *Ec*XynA with XU^4m2^X (PDB ID: http://2Y24). (A) Solvent excluded surface of *Tc*Xyn30B with the XU^4m2^X model derived from *Ec*XynA with XU^4m2^X. Red and blue surfaces show Asn‐93 and Tyr‐209, respectively. (B) Stick and ribbon model of *Tc*Xyn30B with a ligand. A numeric value shows the distance (Å) between Asn‐93 and Tyr‐209. A red circle indicates the hydroxyl group at C‐4 position of Xyl ‐2. An arrow suggests flipping of Asn‐93 accompanied by binding of glucuronoxylan with a high degree of polymerization.

The glucuronoxylanase activity that releases X_n_U^4m2^X from xylan requires the binding of substrate at subsite ‐3 onward. When the *Tc*Xyn30B model was superimposed on the *Ec*XynA model complexed with XU^4m2^X, a steric clash between non‐reducing end of XU^4m2^X and Asn‐93 of *Tc*Xyn30B was observed (Fig. 5A). On the other hand, the structural analysis of *Tc*Xyn30B‐ligand complex revealed that O4‐atom of Xyl ‐2 bound in *Tc*Xyn30B is located at a different position from that bound in *Ec*XynA, oriented toward a groove between Asn‐93 and Tyr‐209 (Fig. 5A,B, red‐dashed circle, and Fig. S5). This suggests that Xyl ‐3 is likely to fit in the groove formed, as predicted previously [11]. However, the distance between Nδ of Asn‐93 and C of Tyr‐209 is calculated to only be 6.2 Å at the narrowest point. Because the van der Waals radii of Cβ of Tyr, C of xylose ‐3 and Oδ of Asn can be considered as 2.0, 1.7 and 1.6, respectively, the distance of the groove should be at least 7.0 Å for binding of Xyl ‐3 [29]. Thus, the groove is too small for Xyl ‐3 to enter spontaneously. From these observations, we propose that a structural change of the groove, such as the flipping of Asn‐93 (Fig. 5B, indicated by an arrow) or a conformational change of the loop, will occur for the binding of substrate with a high degree of polymerization and plays an important role in the switching between xylobiohydrolase and glucuronoxylanase activity. Such a structural change to bind glucuronoxylan may also be facilitated by the strong recognition and orientation of the MeGlcA substituent at ‐2b.

## Conclusions

In the present study, we demonstrated the crystal structure of *Tc*Xyn30B complexed with U^4m2^X and the unique mechanism for substrate recognition in GH30‐7. The structure revealed that *Tc*Xyn30B recognizes not only the C‐6 carboxyl group, but also the 4‐*O*‐methyl group of MeGlcA, unlike GH30‐8 enzymes. Residues interacting with these two functional groups are conserved in GH30‐7 endoxylanases. The enzyme–ligand complex model and site‐directed mutagenesis indicated that the interaction between Asn‐93 on the β2‐α2 loop and Xyl ‐2 residue is partially involved in xylobiohydrolase activity. Our results provide structural insight with respect to substrate recognition in GH30‐7 glucuronoxylanases and xylobiohydrolases.

## Conflict of interest

The authors declare no conflict of interest.

## Author contributions

YN and HI designed the study and mainly contributed to writing the manuscript. YN was responsible for the preparation and crystallization of the proteins. YN and MW performed X‐ray diffraction analysis and processed the data. YN was responsible for modeling and refinement of the crystal structures. AM and HI supervised the study. All authors read and approved the final manuscript submitted for publication.

## Supporting information


**Fig. S1** **.** The structural models and abbreviations of ligands. The shorthand nomenclature used to describe the xylooligosaccharides has been described previously [1].
**Fig. S2** **.** SDS/PAGE analysis of purified *Tc*Xyn30B protein. Lanes: 1, molecular mass standards; 2, purified *Tc*Xyn30B (15 μg protein). The black arrow indicates the position of *Tc*Xyn30B.
**Fig. S3** **.** Crystals of *Tc*Xyn30B complexed with U^4m2^X.
**Fig. S4** **.** *F*_o_‐*F*_c_ omit maps (blue) contoured at 3.0 σ for sugar chains. *N*‐linked carbohydrate moieties. Sugar chains linked Asn‐60, Asn‐88, Asn‐334, Asn‐346 and Asn‐412 are shown. Atoms are coloured as: C of *N*‐linked sugar chain residues, purple; C of *Tc*Xyn30B, brown; O, red; N, blue.
**Fig. S5** **.** Orientations of U^4m2^X bound to *Tc*Xyn30B and XU^4m2^X bound to *Ec*XynA. The model of *Tc*Xyn30B with U^4m2^X was superimposed on *Ec*XynA with XU^4m2^X (PDB ID: https://doi.org/10.2210/pdb2Y24/pdb). The distances are given in Å.

## Data Availability

The crystal structure of *Tc*Xyn30B in complex with and without U^4m2^X have been deposited in the PDB under accession codes http://6KRN and http://6KRL, respectively.
