# Supplementary material for: Substrate recognition by a bifunctional GH30‐7 xylanase B from Talaromyces cellulolyticus
Source: FEBS Open Bio. 2020 May 22;10(6):1180–9. doi: 10.1002/2211-5463.12873 (PMC7262913; doi:10.1002/2211-5463.12873)
Supplement: Supplementary file 1 — Fig. S1 . The structural models and abbreviations of ligands. The shorthand nomenclature used to describe the xylooligosaccharides has been described previously [1]. Fig. S2 . SDS/PAGE analysis of purified TcXyn30B protein. Lanes: 1, molecular mass standards; 2, purified TcXyn30B (15 μg protein). The black arrow indicates the position of TcXyn30B. Fig. S3 . Crystals of TcXyn30B complexed with U4m2X. Fig. S4 . F o‐F c omit maps (blue) contoured at 3.0 σ for sugar chains. N‐linked carbohydrate moieties. Sugar chains linked Asn‐60, Asn‐88, Asn‐334, Asn‐346 and Asn‐412 are shown. Atoms are coloured as: C of N‐linked sugar chain residues, purple; C of TcXyn30B, brown; O, red; N, blue. Fig. S5 . Orientations of U4m2X bound to TcXyn30B and XU4m2X bound to EcXynA. The model of TcXyn30B with U4m2X was superimposed on EcXynA with XU4m2X (PDB ID: https://doi.org/10.2210/pdb2Y24/pdb). The distances are given in Å. [file FEB4-10-1180-s001.pdf]

Substrate recognition by a bifunctional GH30-7 xylanase B from *Talaromyces cellulolyticus*

Yusuke Nakamichi[1], Masahiro Watanabe[1], Akinori Matsushika[1,2], and Hiroyuki Inoue[1]

[1]Research Institute for Sustainable Chemistry, National Institute of Advanced Industrial Science and Technology (AIST), 3-11-32 Kagamiyama, Higashi-Hiroshima, Hiroshima 739-0046, Japan

[2]Graduate School of Advanced Sciences of Matter, Hiroshima University, 1-3-1 Kagamiyama, Higashi-Hiroshima, Hiroshima 739-8530, Japan

Supplementary Information

No.	Contents		Page
1	Figure S1	The structural models and abbreviations of ligands.	S-2
2	Figure S2	SDS-PAGE analysis of purified *Tc*Xyn30B protein	S-3
3	Figure S3	Crystals of *Tc*Xyn30B complexed with $U^{4m2}X$	S-4
4	Figure S4	*F*o-*F*c omit maps (blue) contoured at 3.0 σ for sugar chains	S-5
5	Figure S5	Orientations of $U^{4m2}X$ bound to *Tc*Xyn30B and $XU^{4m2}X$ bound to *Ec*XynA	S-6

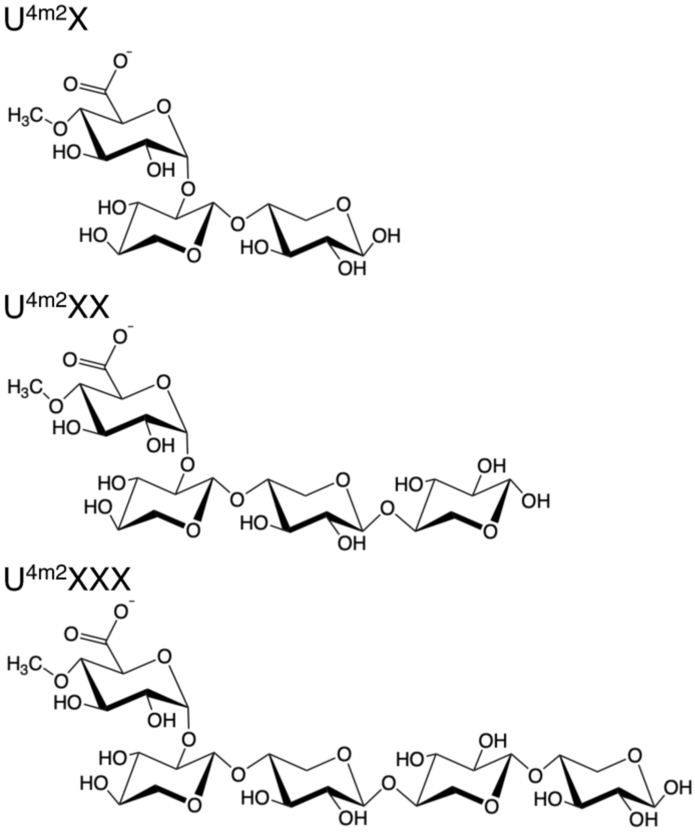

Supplemental Figure S1. The structural models and abbreviations of ligands. The shorthand nomenclature used to describe the xylooligosaccharides has been previously described [1].

Reference

1. Fauré R, Courtin CM, Delcour JA, Dumon C, Faulds CB, Fincher GB, Fort S, Fry SC, Halila S, Kabel MA, Pouvreau L, Quemener B, Rivet A, Saulnier L, Schols HA, Driguez H, O’Donohue MJ. 2009. A brief and Informationally rich naming system for oligosaccharide motifs of heteroxylans found in plant cell walls. Aust J Chem 62:533–537.

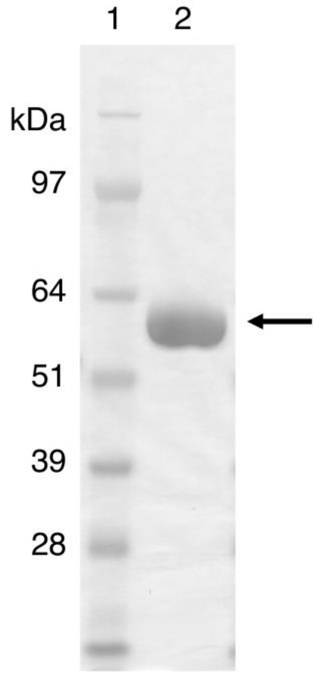

Supplemental Figure S2. SDS-PAGE analysis of purified *Tc*Xyn30B protein. Lanes: 1, molecular mass standards; 2, purified *Tc*Xyn30B (15 μg protein). The black arrow indicates the position of *Tc*Xyn30B.

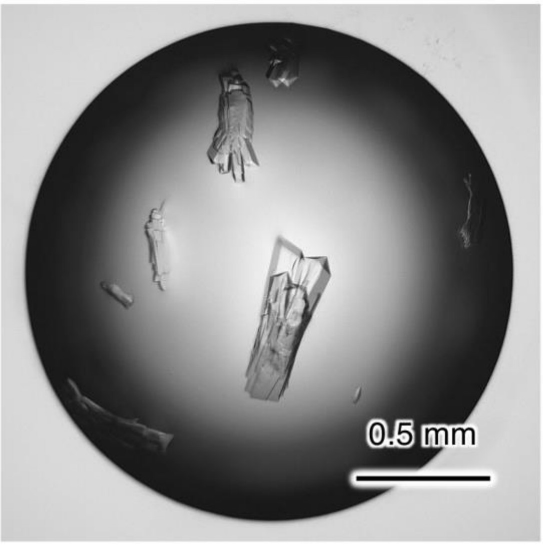

Supplemental Figure S3. Crystals of *Tc*Xyn30B complexed with $U^{4m2}X$.

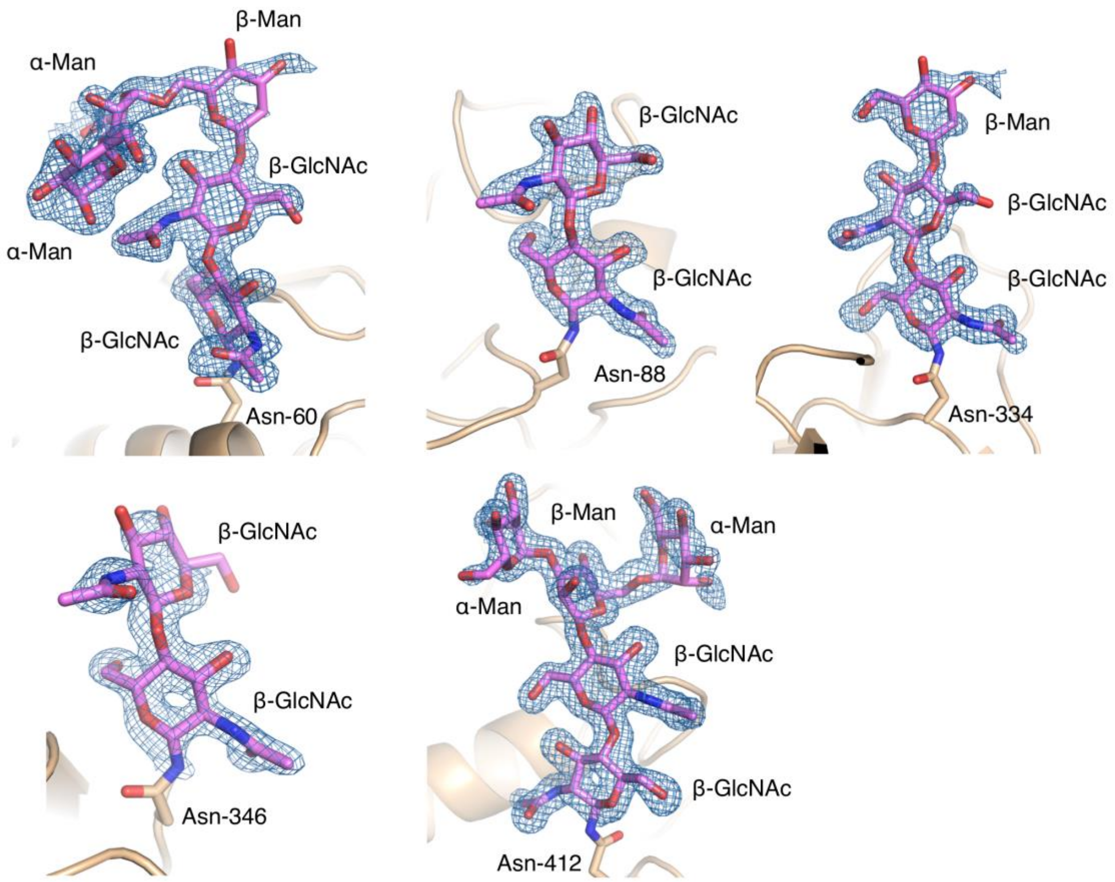

Supplemental Figure S4. *F*o-*F*c omit maps (blue) contoured at 3.0 σ for sugar chains. *N*-linked carbohydrate moieties. Sugar chains linked Asn-60, Asn-88, Asn-334, Asn-346, and Asn-412 are shown. Atoms are coloured as follows: C of *N*-linked sugar chain residues, purple; C of *Tc*Xyn30B, brown; O, red; and N, blue.

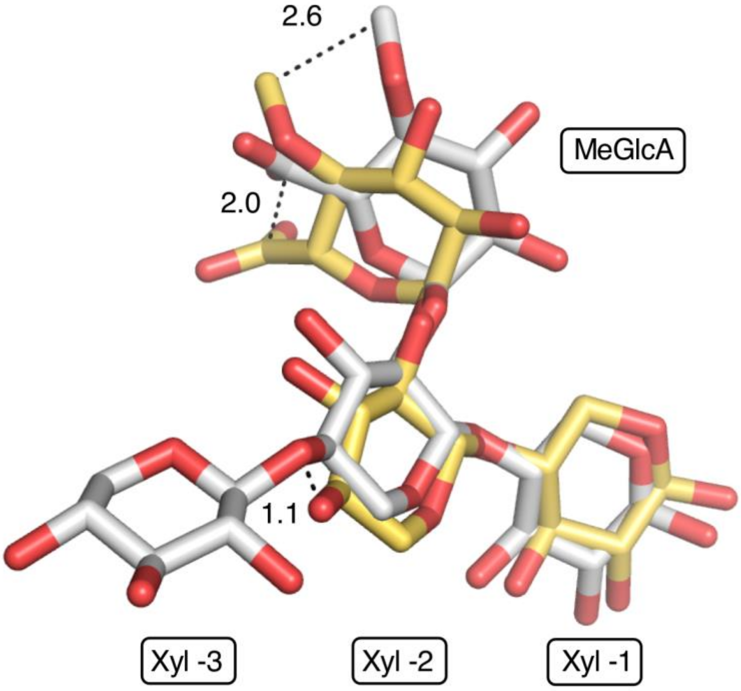

Supplemental Figure S5. Orientations of $U^{4m2}X$ bound to *Tc*Xyn30B and $XU^{4m2}X$ bound to *Ec*XynA. The model of *Tc*Xyn30B with $U^{4m2}X$ was superimposed on *Ec*XynA with $XU^{4m2}X$ (PDB ID, 2Y24). The distances are in Å.
